# Baby schema in human and animal faces induces cuteness perception and gaze allocation in children

**DOI:** 10.3389/fpsyg.2014.00411

**Published:** 2014-05-07

**Authors:** Marta Borgi, Irene Cogliati-Dezza, Victoria Brelsford, Kerstin Meints, Francesca Cirulli

**Affiliations:** ^1^Section of Behavioral Neuroscience, Department of Cell Biology and Neurosciences, Istituto Superiore di SanitàRome, Italy; ^2^School of Psychology, University of LincolnLincoln, UK

**Keywords:** eye-tracking, children, cuteness, gaze pattern, preferential looking, pet animals

## Abstract

The baby schema concept was originally proposed as a set of infantile traits with high appeal for humans, subsequently shown to elicit caretaking behavior and to affect cuteness perception and attentional processes. However, it is unclear whether the response to the baby schema may be extended to the human-animal bond context. Moreover, questions remain as to whether the cute response is constant and persistent or whether it changes with development. In the present study we parametrically manipulated the baby schema in images of humans, dogs, and cats. We analyzed responses of 3–6 year-old children, using both explicit (i.e., cuteness ratings) and implicit (i.e., eye gaze patterns) measures. By means of eye-tracking, we assessed children’s preferential attention to images varying only for the degree of baby schema and explored participants’ fixation patterns during a cuteness task. For comparative purposes, cuteness ratings were also obtained in a sample of adults. Overall our results show that the response to an infantile facial configuration emerges early during development. In children, the baby schema affects both cuteness perception and gaze allocation to infantile stimuli and to specific facial features, an effect not simply limited to human faces. In line with previous research, results confirm human positive appraisal toward animals and inform both educational and therapeutic interventions involving pets, helping to minimize risk factors (e.g., dog bites).

## INTRODUCTION

It has been hypothesized that humans exhibit a natural interest and attraction to other species (the so-called *Biophilia Hypothesis*, Wilson, 1984). A general proneness toward animals is observed in children from a very early stage of development (DeLoache et al., 2011; Lobue et al., 2012; Borgi and Cirulli, 2013). Children are more likely to be attentive and to have increased motivational levels in the presence of animals and this has led to the inclusion of different animal species both in educational and therapeutic interventions aimed at promoting healthy development in children (Cirulli et al., 2011; Endenburg and van Lith, 2011; Berry et al., 2013; O’Haire, 2013). Even in subjects with a deficit in the social domain (i.e., autism spectrum disorder) a preference for animal over human and inanimate stimuli has been shown (Celani, 2002; Prothmann et al., 2009; Grandgeorge et al., 2014) as well as an increase in social behaviors in the presence of animals compared to toys (O’Haire et al., 2013).

Despite recent advances in child psychology research on human–animal interactions (e.g., benefits of contacts with animals during development, dog bite prevention, links between animal and child abuse), very little attention has been paid to the identification of specific animal characteristics underpinning distinct behavioral responses in humans, particularly in children. Preliminary studies analyzing differences in children’s behavior toward robotic, stuffed, and real animals suggest that animal features can impact upon children’s emotional response and willingness to engage in social interactions (Kerepesi et al., 2006; Ribi et al., 2008; Howard and Vick, 2010). However, more research is needed to identify animal features and traits able to influence children’s attraction to animals and, ultimately, their affiliative response toward them.

Most common pet species (i.e., dogs and cats) exhibit both morphological and behavioral infantile characteristics which may have been retained into adulthood as a by-product of the domestication (Belyaev, 1979; Frank and Frank, 1982). This process has been referred to as *neoteny* and it thought to be due to generations of conscious or unconscious selective breeding for non-aggressive behavior toward man (i.e., tameness or docility, Belyaev, 1979). It has been hypothesized that the presence of lifelong youthful traits might form the basis of our attraction to animals, especially pets (Archer, 1997). 

The term *baby schem*a (*Kindchenschema*, Lorenz, 1943) refers to a set of facial features (i.e., large head and a round face, a high and protruding forehead, large eyes, and a small nose and mouth) commonly found both in human and animal infants. In classical ethology this specific configuration of features has been described as triggering an innate releasing mechanism for caregiving and affective orientation toward infants (Lorenz, 1943) and more recently, its role in promoting human nurturing behavior was demonstrated at the neurophysiologic level using neuroimaging (Glocker et al., 2009b). 

Increased attention and willingness to care, positive affect and protective behavior, as well as decreased likelihood of aggression toward the infant, characterize the so-called *baby schema response* or *cute response* (Lorenz, 1943; Alley, 1983; Brosch et al., 2007; Glocker et al., 2009a; Sherman et al., 2009; Nittono et al., 2012). Several empirical studies have employed the use of pictures/drawings to analyze the appeal of the baby schema for humans showing that faces with infantile traits are commonly perceived as cute and attractive and are consistently preferred to those with a less infantile facial configuration (Sternglanz et al., 1977; Hildebrandt and Fitzgerald, 1979; Alley, 1981; Glocker et al., 2009a). Previous research has demonstrated the generalization of this response to real animals (Archer and Monton, 2011; Little, 2012), representations of animals such as cartoon characters (e.g., Mickey Mouse, Gould, 1979) and stuffed/toy animals (e.g., Teddy bear, Hinde and Barden, 1985; Archer and Monton, 2011). Consistent with these observations, the findings of a recent study by Golle et al. (2013) suggest the existence of a common mechanism that codes cuteness of human and non-human infant faces. The idea of the extension of the baby schema response to the human-animal bond context has gained weight also in the light of some evidence that the bond between pets and their owners shares striking similarities to the relationship between human parents and their children, e.g., the language used to talk to animals mimics the so-called *motherese* or *baby talk* (Burnham et al., 2002) and dogs seem to view their owners as a secure base (Horn et al., 2013). 

The analysis of the emergence of a cute response, during development, has so far produced results not easily comparable (Fullard and Reiling, 1976; Maestripieri and Pelka, 2002; Sanefuji et al., 2007; Borgi and Cirulli, 2013). Cuteness perception and preference for infantile features in animals (as well as the pseudo-nurturing behavior toward animal-like toys) seem to emerge in children between 3 and 6 years (Morris et al., 1995; Borgi and Cirulli, 2013). Children’s positive response to the baby schema appears to be influenced by the viewed species, and gender and familiarity with animals (i.e., pet ownership) may modulate preferences (Borgi and Cirulli, 2013).

There are – nonetheless – a range of methodological limitations in the previous findings. First, most of the prior studies have employed drastically simplified stimuli (line drawings and schematic faces) or stimuli not controlled for the individual facial differences unrelated to baby schema (e.g., color, pose, and expression). Hence the interpretation of outcomes is limited by the impossibility to dissociate the response to a specific stimulus (humans vs. animals; adult vs. young) from the response to its facial configuration (i.e., baby schema). Only recently, Glocker et al. (2009a) presented experimental evidence of a baby schema effect in infant faces. This was achieved by developing an effective procedure to create stimuli with objectively quantified and parametrically manipulated baby schema content, that retained all the characteristic of the individual portrait (Glocker et al., 2009a). Second, when asked for overt preferences, participants might only report socially desirable and appropriate responses, as evidenced in traditional self-report measures (e.g., ratings, questionnaires, interviews). Direct preference assessments (such as preferential looking) represent a more reliable and sensitive measure of the observer’s preferences (Fleming et al., 2010) and may shed light on the cognitive mechanisms underlying attraction to different stimuli. In fact, although this aspect is still not extensively explored, the baby schema response seems to be anticipated by an attentional bias toward infantile stimuli. Previous studies have shown a visual prioritization (dotprobe task, Brosch et al., 2007) and a willingness to increase the viewing time to cute images (key-press task, Parsons et al., 2011; Hahn et al., 2013; Sprengelmeyer et al., 2013) in adult participants. In general, adults tend to look longer at infant than at adult faces and at cuter than at less cute infants (Hildebrandt and Fitzgerald, 1978; Power et al., 1982; Parsons et al., 2011; Cárdenas et al., 2013; Hahn et al., 2013; Sprengelmeyer et al., 2013) and women’s interest in and attentional bias toward infants appear to be stronger and more stable than men’s (Cárdenas et al., 2013; Hahn et al., 2013). However, further studies are needed to determine whether this attentional bias is constant and persistent or whether it changes during development. In addition, questions remain as to whether this response may be detected when viewing images of non-human faces.

In order to overcome these limitations, in the present study we have systematically investigated the effects of the baby schema on children’s perception of cuteness in human and animal faces, using both explicit (i.e., cuteness judgment) and implicit (i.e., gaze behavior) measures. We followed Glocker et al.’s (2009a) procedure to create a photographic set of stimuli consisting of facial images of both humans and pets (i.e., dogs and cats), parametrically manipulated to produce two portraits of the same subject: one *high* infantile (round face, high forehead and big eyes, small nose and mouth) and one *low* infantile (narrow face, low forehead and small eyes, big nose and mouth). This procedure allowed us to dissociate the response to the baby schema (high vs. low infantile faces) from the response to different categories of stimuli (humans vs. animals; adult vs. young).

In Experiment 1 we aimed at exploring a possible early emergence of the attentional bias toward the baby schema (Hildebrandt and Fitzgerald, 1978; Power et al., 1982; Parsons et al., 2011; Cárdenas et al., 2013; Hahn et al., 2013; Sprengelmeyer et al., 2013) by evaluating 3–6 years old children’s gaze allocation to infantile images in a preferential looking task. Eye-tracking was used to assess boys’ and girls’ preferential attention to one of two displayed images varying only for the degree of baby schema (side-by-side pictures: high vs. low infantile). 

In Experiment 2 stimuli were individually displayed and children were asked to rate pictures for cuteness (from “not cute” to “very cute”). We aimed at assessing the effect of the baby schema on cuteness perception (Glocker et al., 2009a) and its possible early emergence. Eye-tracking was used to explore participants’ fixation patterns toward displayed images during the cuteness task. Since eye movements can be modulated by cognitive demands and characteristics of the observed scenes (Henderson, 2003; Isaacowitz, 2006), we predicted gaze patterns (gaze distribution across key internal facial features, i.e., eyes, nose, and mouth) to be sensitive to cues specifically related to infant-like characteristics, and are the first to test such an assumption in children. 

Children’s ratings were compared to those given by a sample of adult participants (Experiment 2) and the effect of previous experience with animals (i.e., pet ownership) in modulating both cuteness perception and attentional responses assessed (Experiment 1 and 2). In fact, although most of the previous research has described the baby schema response as driven by baby-like perceptual factors (i.e., bottom-up process), recent studies have shown that there are also top-down processes at play (Kaufman et al., 2013). The effect of facial features on cuteness perception may thus be modulated by motivations, preferences and prior experience, e.g., relationship with a child or age group (Kaufman et al., 2013), interest in infants and motivation to care (Light and Isaacowitz, 2006; Cárdenas et al., 2013), pet ownership and attachment to pets (Archer and Monton, 2011; Borgi and Cirulli, 2013). 

## MATERIALS AND METHODS

### STIMULUS CREATION

#### Overview

The stimuli were based on a set of 120 color photographs (full frontal view, looking at camera, closed mouth, and neutral facial expression) depicting 20 faces for each of the following six categories: human adults, human infants, adult dogs, puppies, adult cats, kittens. Most of the images were obtained from Thinkstock/Getty Images^1^ (courtesy of Gioacchino Altamura) and some were collected by submitting key-words like “Infant face” or “Dog face” to Google Images web search engine. Following Glocker et al.’s (2009a) method, pictures were modified to produce faces with parametrically manipulated baby schema consisting of high and low infantile features for each portrait. In particular, in Glocker’s study baby schema was operationalized using facial features that had previously been suggested to contribute to the baby schema response (and recognized as typical anatomical infant characteristics) such as face width, forehead length and eye, nose, and mouth size. Baby schema content in each image was manipulated using the range of baby schema values (mean and standard deviation, SD) in a sample of unmanipulated images as a guide for the manipulation procedure which consisted in reducing or enlarging facial parameters (above or below the mean; with the range of manipulation depending on SD) to produce either high or low infantile features (Glocker et al., 2009a). In our study, Glocker’s method, originally developed to modified human infant faces, was for the first time applied also to faces of human adults and to those of adult and young animals (dogs and cats).

#### Stimulus creation procedure

**FIGURE 1:**
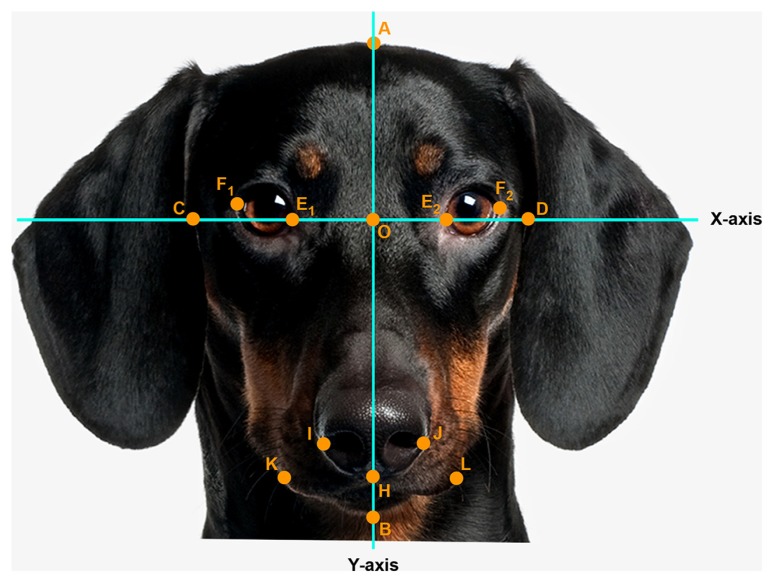
**Facial landmark (example: portrait of an adult dog).** Head length (AB, fixed, 600 pixels), face width (CD), forehead length (AO), eye width (EF, as the average calculated from the right, E_1_F_1_ and left E_2_F_2_ eye width), nose length (OH), nose width (IJ), mouth width (KL). Photo: Thinkstock/Getty Images (modified).

Pictures were digitized at 72 dpi and were two-dimensionally rotated and scaled to a head length of 600 pixels. A coordinate system was superimposed on the faces so that the *x*-axis connected the inner corner of the eyes and the *y*-axis traversed the midline of the nose. Facial measurements were obtained by measuring distances between the following landmarks (see **Figure 1**): A (top of the head), B (bottom of the chin), C and D (outer edges of the face along the *x*-axis), E1 and E2 (inner corners of the eyes), F1 and F2 (outer corners of the eyes), O (nose base at the crossing of the *x*- and *y*-axis), H (below the tip of the nose), I and J (widest point on nose wing), K and L (outer edge of the mouth).

Using Adobe Photoshop ruler tool with pixel as unit, the following distances between landmarks were measured (**Table 1**, column A): head length (AB, fixed, 600 pixels), face width (CD), forehead length (AO), eye width (EF, as the average calculated from the right, E_1_F_1_ and left E_2_F_2_ eye width), nose length (OH), nose width (IJ), mouth width (KL). The baby schema was captured by six facial parameters (**Table 1**, column B): CD as an absolute measure in pixels with reference to the head length of 600 pixels, and five proportion indices (relative size of one facial measure to another): AO/AB, EF/CD, OH/AB, IJ/CD, KL/CD. The mean and standard deviation for each facial parameter was calculated from the sample of 20 unmanipulated images for each category (**Table 1**, column C). These values served as a guide for our manipulation (normalized mean values; *z*-scores; Glocker et al., 2009a).

**Table 1:** Measurements taken (A) and baby schema facial parameters (B) in a sample of 20 unmanipulated images (mean and SD; C).

		Mean (SD) (C)					
Measurements (A)	Facial parameters (B)	Human adult	Human infant	Dog	Puppy	Cat	Kitten
AB = head length (fixed)	–	600	600	600	600	600	600
CD = face width	CD	365.0 (16.0)	391.6 (23.5)	436.1 (66.5)	479.9 (91.0)	589.3 (44.4)	688.3 (48.4)
AO = forehead length	AO/AB	0.48 (0.02)	0.61 (0.03)	0.44 (0.06)	0.49 (0.05)	0.56 (0.03)	0.59 (0.05)
EF = eye width (average)	EF/CD	0.17 (0.01)	0.19 (0.01)	0.12 (0.03)	0.12 (0.02)	0.16 (0.01)	0.15 (0.01)
OH = nose length	OH/AB	0.21 (0.02)	0.14 (0.01)	0.42 (0.09)	0.34 (0.07)	0.29 (0.02)	0.23 (0.03)
IJ = nose width	IJ/CD	0.26 (0.02)	0.24 (0.02)	0.30 (0.04)	0.23 (0.06)	0.15 (0.02)	0.13 (0.01)
KL = mouth width	KL/CD	0.35 (0.02)	0.29 (0.04)	0.62 (0.16)	0.61 (0.14)	0.41 (0.07)	0.35 (0.06)

*Standard deviations are given in parentheses.*

Using Adobe Photoshop we then manipulated these facial parameters to produce high (round face, high forehead and big eyes, small nose, and mouth; CD, AO/AB and EF/CD > mean; OH/AB, IJ/CD and KL/CD < mean) and low (narrow face, low forehead and small eyes, big nose and mouth; CD, AO/AB, and EF/CD < mean; OH/AB, IJ/CD and KL/CD > mean) infantile portraits of each subject. Photoshop resize tool on masked layers (which allow to modify a particular facial feature without affecting the others) was used to enlarge or reduce (in order) forehead length, nose length, face width, eye width, nose width, and mouth width; clone stamp and healing brush tools were used to adjust sections of the picture which appeared unnaturally stretched. To maintain normal facial appearance, the manipulation for each facial parameter was restricted to a *z*-score range of ±2 standard deviations (Geldart et al., 1999; Glocker et al., 2009a). Since unmanipulated faces often combined high and low infantile features, only those parameters which needed an adjustment were manipulated (Glocker et al., 2009a).

**FIGURE 2:**
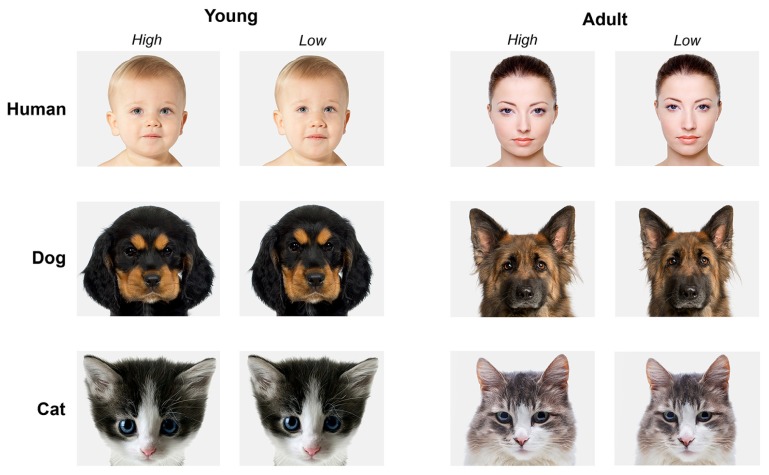
**Examples of stimuli presented to participants.** Young and adult faces of humans, dogs, and cats. On the left the *high* infantile version, on the right the *low* infantile version of the same subject. Photos: Thinkstock/Getty Images (modified).

Using this protocol, a sub-set of 24 pictures (four different portraits for each category: human adults, human infants, adult dogs, puppies, adult cats, kittens) was manipulated. Picture selection was made on the basis of image quality, uniform background, clarity of facial expression. This resulted in a set of 48 faces consisting of 24 high and 24 low infantile portraits. Image background was set to 5% gray. Brightness of the pictures was visually adjusted to appear similar between all pictures (see **Figure 2** for examples).

### EXPERIMENT 1

#### Participants

Fifty (27 boys and 23 girls) British children, ranging in age between 3 and 6 years (*M* = 4.5, SD = 1.0) participated in this experiment. Children were recruited in schools (four nursery schools and one primary school in Lincoln and Lincolnshire, UK). All children had normal or corrected-to-normal eyesight. Children were only excluded if they had a certified developmental disability or were unwilling to participate spontaneously. Informed consent was obtained first by schools and nurseries and then from parents, in line with the British Psychological association guidelines. Children were also asked to give their assent and were not tested if they did not want to take part. All children and all parents were aware that they could withdraw at any time. The University of Lincoln Human Research Ethics Committee approved the protocol and procedure. Parents of participating children received a questionnaire comprising children’s demographic information and presence of animals at home. Questionnaires were distributed by email or hand-delivered to parents. Nineteen of the participants were dog owners, 11 cat owners and 24 did not own pets.

#### Visual stimuli

Stimuli were presented as two side-by-side pictures, one showing a high infantile and the other a low infantile version of the same subject (total: 24 pairs of images - 4 different subjects per 6 face categories, i.e., human adults, human infants, dogs, puppies, cats, kittens). The size of each picture was 400 × 290 pixels, and the distance between the two images was 224 pixels. The order of presentation was randomized, and the presentation of the images was counterbalanced to appear on the left and right side of the screen equally often (given children’s tendency for left gaze preference, Guo et al., 2009).

#### Procedure

Participants’ eye movements were tracked at a sample rate of 120 Hz using a Tobii T120 eye tracker (Tobii, Stockholm, Sweden), which is integrated into a 17′′ TFT monitor (pictures displayed with a resolution of 1024 × 768 pixels). Each child was tested individually in an intermodal preferential looking (IPL) task (see Meints et al., 1999, 2002 for details on IPL procedure). Children were asked if they would like to look at some pictures on a screen, and if they agreed, they were first familiarized with a quiet testing room for a few minutes. Children sat at a distance of approximately 70 cm from the monitor. At this distance the eye-tracking device allows free head movement in a wide operating range (30 cm × 22 cm × 30 cm). Before testing, the child’s vision was centered to the middle of the screen and the eye tracker was calibrated using five fixation points (FPs). After the calibration procedure, the experiment began with a short introduction in which children were instructed to look at the images. Each trial was started with a small red FP displayed on the center of the monitor to attract the child’s gaze to the center of the screen (between the two images). Once the child’s gaze was oriented toward the FP a visual stimulus was presented for 6 s and during this time window eye position was recorded. The onset of the image presentation was accompanied by a female auditory instruction to “look” delivered through a loudspeaker positioned centrally below the displayed pictures. Inter-trial intervals varied with the child’s attention on the task because a new trial was not launched until children were attracted by the FP (minimum inter-stimulus interval 1 s). Two experimenters tested all children, one behind the screen, not visible to the children, controlling trial onset and monitoring children’s eyes through a laptop connected to the eye-tracker, the other sitting centrally behind the child encouraging him/her to keep their head still. Children were encouraged to accomplish the task and received a small gift and a sticker in return for participating in the study. The total testing time for each child did not exceed 10 min. 

#### Data analysis and statistics

Participants’ eye movements were analyzed using the Lincoln Infant Lab Package software (Meints and Woodford, 2008): fixations (periods of relative gaze stability, Henderson, 2003) were extracted from the raw eye movement data, using dispersion threshold (maximum fixation radius = 1) and duration threshold (minimum fixation duration = 100 ms) criteria (Salvucci and Goldberg, 2000). The number of fixations and associated viewing time (sum of individual fixation durations) directed at each image was computed. Two mixed-model ANOVAs (dependent variables: number of fixations and viewing time) were carried out with Images’ Species (human, dog, and cat), Age (adult and young), and Baby schema (high and low) as within-subjects factors, and Gender and Pet ownership as between-subjects factors.

### EXPERIMENT 2

#### Participants

***Children.*** Thirty-two (16 male and 16 female) British children, ranging in age between 3 and 6 years (*M* = 4.8, SD = 1.0) participated in this experiment. None of these children participated in Experiment 1. Exclusion criteria and recruitment, data collection and ethics procedures were the same as in Experiment 1. Fifteen of the participants were dog owners, 6 cat owners and 14 did not own pets.

***Adults.*** Fifty-eight (48 female and 10 male) undergraduate students with a mean age of 21 years (age range = 18–47 years, SD = 5.9) were recruited during a Psychology course (University of Lincoln, UK) and were asked to participate in a study involving voluntary completion of an anonymous, web-based questionnaire. Only participants with British nationality and who completed the questionnaire were included in data analysis; participants who had children were excluded. Seventeen of the participants were dog owners, 20 cat owners and 26 did not own pets.

#### Visual stimuli

Stimuli consisted of 24 images (four different subjects per six face categories – human adults, human infants, dogs, puppies, cats, kittens) which were displayed individually in the center of the monitor (pictures dimension = 600 × 430 pixels). Two of the four images for each category showed high infantile and two low infantile faces (counterbalanced between participants). Order of picture presentation was randomized. 

#### Procedure

***Children.*** Apparatus and calibration procedure were the same as in Experiment 1. After the calibration procedure, the experiment began with a short introduction in which children were instructed to look at the images and to rate how cute they found each picture on a 5-point scale, with 1 representing “not cute” and 5 “very cute.” Children were presented with a visual analog rating scale (modified from Ernst et al., 2000) depicting five human silhouettes indicating increasing quantities using hand gestures; the scale thus relies on children’s natural way to express quantities. In order to assess whether children understood the notion of cuteness, we first asked them to provide us with some definition of the word “cute” and only children who showed a clear understanding of the notion of “being cute” were included in data analysis (the most common accepted definition children gave us were “pretty,” “nice,” “small,” “fluffy,” and “adorable”). The experimenter then explained the scale and asked the child to explain it back to her to check the child’s understanding. All children understood the scale and the task. Participants were then presented with a random sequence of the stimuli. Each trial showed the visual stimulus for 6 s (during this window eye position was recorded), then the rating scale appeared on the monitor and after children gave us their rating of the picture, the next stimulus appeared. Children could give their response either verbally (by telling us how cute they found the picture, e.g., “very cute”) or by pointing to the correspondent silhouette on the scale visible on the monitor. The onset of the image presentation was accompanied by a female voice auditory instruction to “look” delivered through a loudspeaker positioned centrally below the displayed pictures. The inter-trial interval varied depending on the time children needed for the rating. Two experimenters tested all children, as in Experiment 1. Participants were encouraged to accomplish the task and received a small gift and a sticker in return for participating in the study. The total testing time for each child did not exceed 10 min.

***Adults.*** Participants were asked to complete an anonymous, web-based questionnaire created in Qualtrics^2^ (Qualtrics, Provo, UT, USA). The questionnaire consisted of two sections: (1) a personal details section, comprising information on sex, age, nationality, parenthood, presence of animals at home; (2) a series of photographs to be rated for cuteness (pictures presented were identical to those viewed by children in the eye-tracking experiment).

#### Data analysis and statistics

***Cuteness ratings (children vs. adults).*** Scores given to the different categories of faces (human adult, infant, dog, puppy, cat, and kitten) were averaged, separately for the modified *high* and *low* infantile versions, to give 12 mean scores for each participant. A mixed-model ANOVA was carried out (see Experiment 1) with Participant’s Type (children, adults) entered as additional between-subjects factor (a non-parametric analysis was also performed, showing similar results).

**FIGURE 3:**
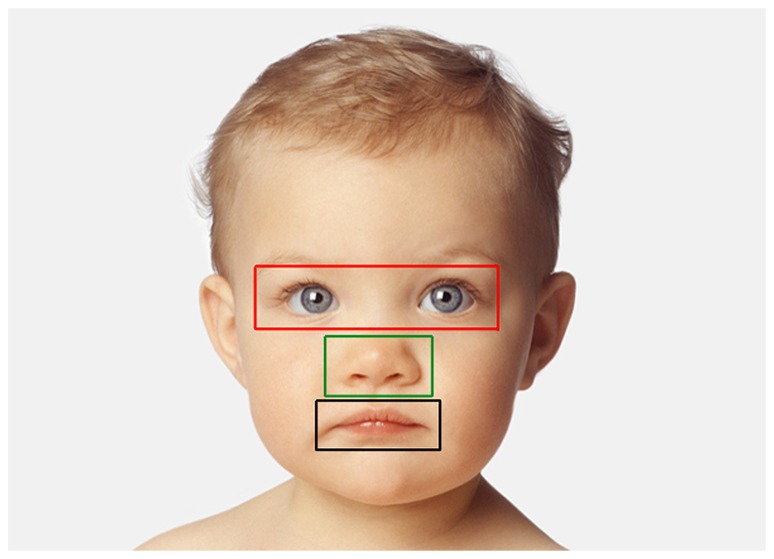
**Example of AOIs for one face (human infant).** AOIs include three separate feature regions: eye (red), nose (green), and mouth (black).

***Looking data (children).*** The number of fixations and associated viewing time (sum of individual fixation durations) directed at each image was computed. We then divided each picture presented into three areas of interest (AOIs) corresponding to three key internal facial features: eyes, nose, and mouth. Specifically, AOIs consisted of centered squares of different dimensions delimiting each possible facial feature and the immediate surrounding area (10 pixels were added in each direction – left, top, right, bottom – with the exception of the division line between the “mouth” and “nose” region: in this case the division line was the midline between the upper lip and the bottom of the nose; **Figure 3**). Each fixation was then characterized by its location among AOIs and the number of fixations (and associated viewing time) directed at each facial feature was normalized, respectively to the total number of fixations and viewing time in that trial. As the same facial features across faces of different species and individuals vary in size, we adopted the criteria from Guo et al. (2010) that consist of subtracting the proportion of the area of each facial feature relative to the whole image from the proportion of fixations (or proportion of total viewing time) directed at that facial feature in a given trial. Any difference in fixation distribution from zero means that this particular facial feature attracted more or less fixations than predicted by a uniform looking strategy, thus negative values demonstrate less viewing than predicted by region size, and positive values demonstrate more viewing than predicted by region size (Dahl et al., 2009; Guo et al., 2010). Two mixed-model ANOVAs (dependent variables: number of fixations and viewing time) were carried out (see above) with AOIs entered as additional within-subjects variable.

## RESULTS

### EXPERIMENT 1 (CHILDREN)

**FIGURE 4:**
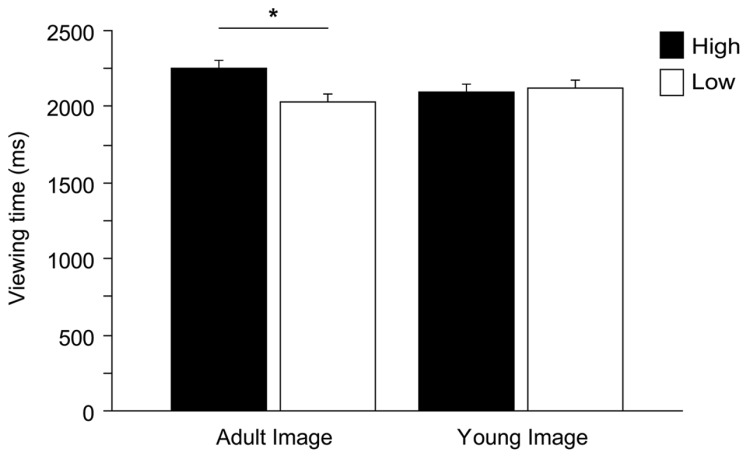
**Children’s preferential looking.** Viewing time (ms) directed to high and low versions of images depicting adult and young faces. ANOVA followed by Tukey *post hoc* test, *p < 0.05. All data are shown as mean + SEM.

Within a 6-s presentation time, on average participants dedicated 4.3 ± 1.2 s (mean ± SD) to explore stimuli. We observed that an excessively short looking time in a trial was generally caused by momentary distraction (children looking elsewhere, i.e., looking at the experimenters or objects in the room). We therefore removed those trials with a total looking time shorter than 1 s (2.3% of the trials). Two ANOVAs (dependent variables: number of fixations and viewing time, see above) were run to test against the null hypothesis of equal distribution of gaze between high and low infantile pictures. Gender and Pet ownership were eliminated from the model (neither main effects nor interaction effects found, viewing time all *F* < 2.669, all *p* > 0.109; fixations all *F* < 1.784, all *p* > 0.188). ANOVAs show that, on average, children allocated significantly longer viewing time to the high infantile pictures, independently of the species [main effect Baby schema, *F*_(1,49)_ = 5.272, *p* = 0.026; interaction effect Species^*^Baby schema, *F*_(2,98)_ = 0.163, *p* = 0.850], while no significant differences were found in the number of fixations allocated to high and low pictures [Baby schema, *F*_(1,49)_ = 2.334, *p* = 0.133]. Children’s preferential looking to high infantile images was driven primarily by adult images with the effect approaching significance [Baby schema^*^Adultness, *F*_(1,49)_ = 3.889, *p* = 0.054]. In particular, while high infantile adult faces were looked at longer than low infantile adult faces (Tukey *post hoc* tests, *p* < 0.05), no differences were found when analyzing the viewing time allocated to high and low infantile images of young faces (*p* > 0.05; **Figure 4**).

### EXPERIMENT 2

#### Cuteness ratings (children vs. adults)

Averaged scores given to the different categories of faces (human adult, infant, dog, puppy, cat, and kitten), separately for the modified *high* and *low* infantile versions, are shown in **Table 2**. 

**Table 2:** Averaged cuteness ratings for both high infantile and low infantile versions of each image category, given by children and adult participants.

	Human adult		Human infant		Dog		Puppy		Cat		Kitten	
	High	Low	High	Low	High	Low	High	Low	High	Low	High	Low
*Children* (*n* = 32)	2.7 (1.3)	2.5 (1.0)	3.5 (1.1)	3.2 (1.2)	3.7 (1.2)	3.7 (1.1)	3.8 (1.1)	3.5 (1.2)	3.6 (1.2)	3.5 (1.1)	3.9 (1.0)	3.6 (1.1)
*Adults* (*n* = 58)	2.4 (0.9)	2.2 (0.7)	3.6 (1.1)	3.0 (1.1)	3.6 (0.9)	3.5 (0.9)	4.1 (0.9)	4.0 (0.9)	3.0 (1.0)	2.7 (1.0)	3.8 (0.8)	3.9 (0.9)

*Standard deviations are given in parentheses.*

**FIGURE 5:**
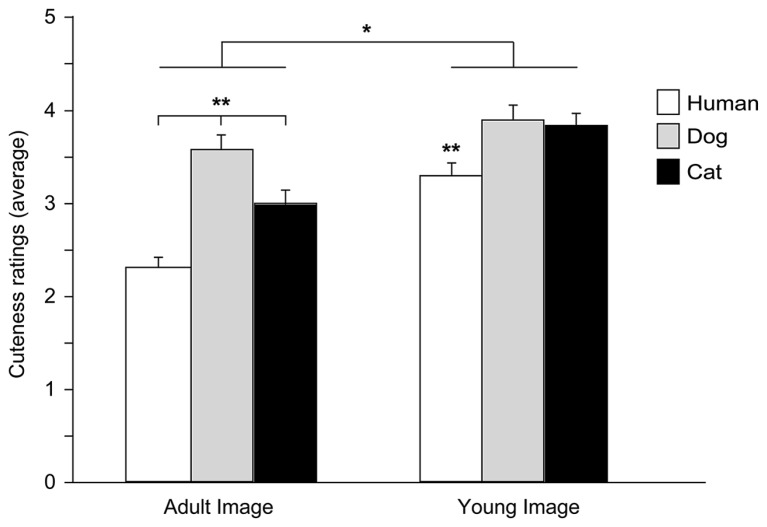
**Cuteness ratings.** Average cuteness ratings given to images of adult and young faces of three species (human, dog, and cat). ANOVA followed by Tukey *post hoc* test, Adult dog > cat > human; Young dog = cat > human, **p < 0.01; Young images > Adult images, *p < 0.05. All data are shown as mean + SEM.

An ANOVA was carried out with Species (human, dog, and cat), Age (adult and young), and Baby schema (high and low) as within-subjects factors and Participant’s Type (children, adults) and Pet ownership as between-subjects factors (a preliminary ANOVA showed no effects of Gender, all *F* < 2.444, all *p* > 0.090, thus this variable was eliminated from the model). A significant main effect of Image’s Species [*F*_(2,172)_ = 36.148, *p* = 0.000] and Age [*F*_(1,86)_ = 101.500, *p* = 0.000] and a significant interaction effect between Species and Age [*F*_(2,172)_ = 12.170, *p* = 0.000] were found. In particular, when rating images of adult portraits, participants gave the highest score to dog faces, followed by the cat and then the human faces (Tukey *post hoc* tests *p* < 0.01), while images of puppies and kittens received a similar score (but higher than human infants, *p* < 0.01); faces of young individuals were rated as cuter than those of adults in all species viewed (all *p* < 0.05; **Figure 5**).

Facial modification for the degree of baby schema had a significant effect on participants’ cuteness judgments. On average participants rated *high* infantile faces as cuter than *low* infantile faces [main effect of Baby Schema, *F*_(1,86)_ = 12.427, *p* = 0.001], an effect independent of the species viewed [Species^*^Baby schema: *F*_(2,172)_ = 1.684, *p* = 0.189] and particularly pronounced in pet owners [Baby Schema^*^Pet Ownership: *F*_(2,56)_ = 7.007, *p* = 0.001]. On average, cuteness scores given by children and adult participants did not significantly differ [main effect of Participant type: *F*_(1,86)_ = 0.901, *p* = 0.345], except when judging images of subjects of different age (adult vs. young faces). Children’s scores given to young and adult faces were not significantly different, while adult participants judged faces of young individuals as cuter than those of adult subjects [Image’s Age^*^Participant’s type: *F*_(1,86)_ = 27.153, *p* = 0.000].

#### Looking data (children)

Participants dedicated on average 4.0 ± 1.1 s (mean ± SD) to explore images within a 6-s presentation time. As above, trials with a total looking time shorter than 1 s (3.7% of the trials) were removed from the analysis. Preliminary analyses showed no effects of gender and pet ownership (viewing time all *F* < 2.658, all *p* > 0.116, fixations all *F* < 2.327, all *p* > 0.108), thus these variables were eliminated from the model. Two mixed-model ANOVAs (dependent variables: number of fixations and viewing time) were carried out with Species (human, dog, and cat), Age (adult and young) and Baby schema (high and low) as within-subjects factors. No effect of Species was observed: number of fixations and associated viewing time per image across human, dog and cat faces did not significantly differ [viewing time, *F*_(2,5__8__)_ = 2.812, *p* = 0.068; fixations: *F*_(2,58)_ = 2.703, *p* = 0.076]. Participants allocated more overall viewing time to images of young faces [*F*_(1,2__9__)_ = 5.099, *p* = 0.032], while showing no differences in the number of fixations [*F*_(1,2__9__)_ = 1.899, *p* = 0.179]. No effects were found for degree of baby schema [high vs. low; fixations: *F*_(1,2__9__)_ = 0.070, *p* = 0.409; viewing time: *F*_(1,2__9__)_ = 0.007, *p* = 0.9325]. 

**FIGURE 6:**
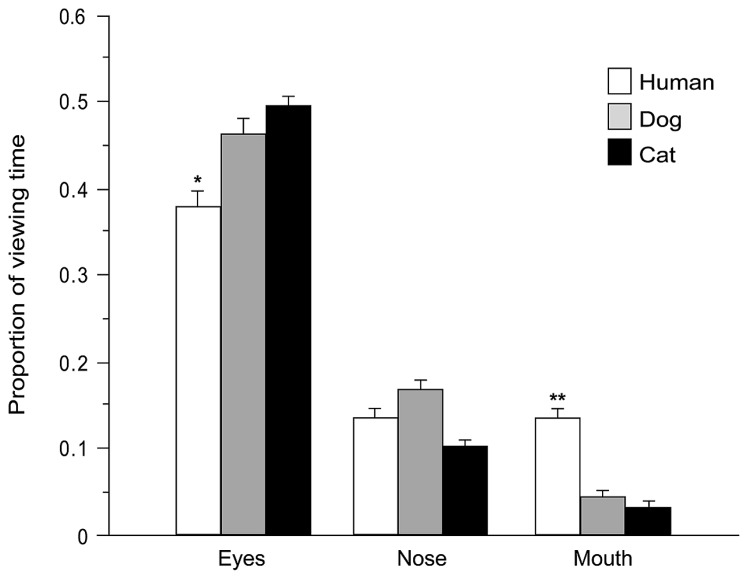
**Species-specific gaze distribution among areas of interest.** Proportion of viewing time directed at eyes, nose, and mouth regions (AOIs) of different species (human, dog, and cat faces). ANOVA followed by Tukey *post hoc* test, **p < 0.01 vs. dog and cat; *p < 0.05 vs. dog and cat. All data are shown as mean + SEM.

During face exploration children directed the majority of fixations (75% of overall fixations) and viewing time (78% of total face viewing time within a trial) at the predefined AOIs (eyes, nose, and mouth). Number of fixations and viewing time directed at each AOI were expressed as proportion of total number of fixations and viewing time within whole faces (after subtracting the proportion of the area of each AOI relative to the whole image, in order to adjust for the variance in size across different stimuli, Guo et al., 2010). Two mixed-model ANOVAs were carried out (see above) with AOI (eyes, nose, mouth) entered as additional within-subjects factor. Highly significant main effects of AOI were found [fixations: *F*_(2,5__8__)_ = 115.645, *p* = 0.000, viewing time: *F*_(2,5__8__)_ = 92.354, *p* = 0.000]. Tukey *post hoc* tests (*p* < 0.01) demonstrate that, irrespective of the face viewed [no main effects of Species or Age or Baby schema were found, all *F* < 2.321, all *p* > 0.05], the eyes attracted the highest proportion of fixations (46%) and the longest viewing time (47%), followed by the nose (fixation and viewing time 13%) and the mouth (fixation 6%, viewing time 7%). However, the significant interactions found between Species and AOI [fixations: *F*_(4,1__16__)_ = 14.778, *p* = 0.000; viewing time: *F*_(4,116)_ = 12.840, *p* = 0.000], Age and AOI [*F*_(2,5__8__)_ = 3.111, *p* = 0.052 and *F*_(2,58)_ = 8.368, *p* = 0.001], and Baby schema and AOI [*F*_(2,5__8__)_ = 6.028, *p* = 0.004 and *F*_(2,58)_ = 6.535, *p* = 0.003] showed that the amount of viewing time allocation to the same facial feature was species-dependent and was also sensitive to the degree of infant features. Specifically, Tukey *post hoc* tests revealed that the eyes attracted a higher proportion of fixations and viewing times in dogs and cats than in human faces (*p* < 0.05), while the mouth attracted a lower proportion of fixations and viewing times in dogs and cats than in human faces (*p* < 0.01; **Figure 6**). Moreover, children directed a significantly higher proportion of viewing time toward the eye region in images of *high* infantile individuals, in comparison with the eyes region in images of *low* infantile individuals (*p* < 0.05).

## DISCUSSION

In this study, the effect of the baby schema on cuteness perception and preferential looking was for the first time assessed in young children (3–6 years old) using eye-tracking techniques and a controlled design in which stimuli (human and animal faces) were objectively quantified according to the baby schema content. The procedure used to modify facial configurations was originally developed by Glocker et al. (2009a) and applied to faces of human infants to show that the baby schema affects cuteness perception and motivation for caretaking in adults, also suggesting a neurophysiologic mechanism by which baby schema could promote human nurturing behavior (Glocker et al., 2009b). 

Overall our results show that the response to an infantile facial configuration can be detected early during development. In children, the baby schema influences both cuteness perception and gaze allocation to infantile stimuli and to specific facial features, an effect not simply limited to human faces.

### VISUAL PREFERENCE FOR BABY SCHEMA

There is evidence that adults tend to look longer at infant than at adult faces and at cuter than at less cute infants (Hildebrandt and Fitzgerald, 1978; Power et al., 1982; Parsons et al., 2011; Cárdenas et al., 2013; Hahn et al., 2013; Sprengelmeyer et al., 2013). The emergence of this attentional response during development, and its generalization to non-human faces, is largely unknown. Our results provide the first rigorous demonstration that a visual preference for baby schema emerges very early during development. Independently of the species viewed, children in our study allocated overall more viewing time to images with a higher degree of baby schema (high vs. low infantile). Our findings contrasts with those of Brosch et al. (2007) who found that the attentional capture by baby schema pictures was specific for human stimuli, a result which authors interpreted as reflecting an adjustment of the human brain to the perception of conspecifics. However, it should be taken into account that Brosch’s study investigated allocation of attention among adults using a very different approach (i.e., dot probe paradigm). In our study visual preference for high infantile stimuli was more pronounced when viewing pictures of adult faces, while children appeared not to be able to discriminate between images of young faces with a different degree of baby schema. Most of previous studies have utilized images of different subjects (infants vs. adults or stimuli previously judged for cuteness), thus with great variation in their appearance, i.e., age, expression, cuteness (Hildebrandt and Fitzgerald, 1978; Power et al., 1982; Parsons et al., 2011; Cárdenas et al., 2013; Sprengelmeyer et al., 2013). Employing image manipulation techniques to alter the facial configuration more subtly, it was shown that the salience of infant stimuli may depend on the ability to evaluate small differences (Sprengelmeyer et al., 2009; Lobmaier et al., 2010), a capacity that may emerge later in life. This observation was also confirmed by the failure to detect gender effects in children, a result that contrasts with what was previously observed in adults (i.e., women showing an higher cuteness sensitivity, Sprengelmeyer et al., 2009; Lobmaier et al., 2010). However, we cannot exclude that the novelty of seeing baby-faced adults may have had an effect on children who looked longer at those faces. Future studies should employ visual stimuli in which the degree of baby schema varies gradually (e.g., 0, 25, 50, 75, 100%), in a sample more representative of the different stages of development (children, adolescent, adults). Preliminary tests aimed at assessing possible novelty effects are also recommended in order to validate the stimuli employed. 

### BABY SCHEMA AND CUTENESS PERCEPTION

Our results show that the degree of baby schema drives cuteness perception in young children: independent of the species viewed, overall faces with a modified infantile facial configuration (round face, high forehead and big eyes, small nose and mouth) were perceived as cuter than those with less infantile traits. Compared to previous studies which have shown the effectiveness of the baby schema on cuteness perception in an adult population (Glocker et al., 2009a) our study extends these findings to young children. Our results are also consistent with previous studies that, using non parametrically manipulated stimuli, showed a generalization of such a response to real animals and representations of animals (Fullard and Reiling, 1976; Gould, 1979; Hinde and Barden, 1985; Maestripieri and Pelka, 2002; Sanefuji et al., 2007; Archer and Monton, 2011; Little, 2012; Borgi and Cirulli, 2013) and support the recent claim of the existence of a common processing mechanism that underlies cuteness perception across species (Golle et al., 2013). 

By applying Glocker’s procedure, we were able to dissociate the response to a specific stimulus (e.g., humans vs. animals) from the response to its facial configuration (i.e., high vs. low baby schema). Independently of the degree of baby schema, adult and children in our study showed a more positive appraisal for animal than for human stimuli, and, among animals, they gave the highest score to the dog followed by the cat (an effect that disappeared when viewing young faces: puppies and kittens received a similar score). Humans’ positive response toward animals (e.g., preference for animal over inanimate and human stimuli, positive behaviors directed to animals), as well as the highest rate of the dog, were previously shown in a number of studies (DeLoache et al., 2011; Lobue et al., 2012; Borgi and Cirulli, 2013). Our results suggest that the appeal of infantile features only partially explains why animals have a powerful hold over human perception. Attitudes and preferences, as well as experiential factors, may have affected participants’ judgment, as was previously shown in a large population of children of the same age (Borgi and Cirulli, 2013). Most of previous investigations have described the baby schema response as a bottom-up process exploring the effect of infantile perceptual features in eliciting a positive emotional reaction and in turn motivation to care. Recent experimental evidence have also emphasized the role of top-down processes in the baby schema response showing how the effect of facial appearance on cuteness and attractiveness may be tied to human interest in infants and motivation to care (Light and Isaacowitz, 2006; Cárdenas et al., 2013), as well as prior experience with infants and infant-like animals (Archer and Monton, 2011; Borgi and Cirulli, 2013; Kaufman et al., 2013).

In the current study pet owners showed to better discriminate differences in infantile facial traits than participants who did not own pets, suggesting a possible effect of experience in the emergence of a “cuteness sensitivity” (the infantile facial configuration in pets is retained into adulthood, Belyaev, 1979; Frank and Frank, 1982). By contrast, no gender effects were found either in children or in the sample of adult participants. Although data are still conflicted (Glocker et al., 2009a; Parsons et al., 2011), a number of studies have shown women to be more responsive to the baby schema then men: they tend to prefer baby-like stimuli and appear more motivated to exhibit nurturing behavior than men (Sternglanz et al., 1977; Hildebrandt and Fitzgerald, 1979; Alley, 1983; Maestripieri and Pelka, 2002; Sprengelmeyer et al., 2009; Lobmaier et al., 2010; Cárdenas et al., 2013). However, it should be taken into account that in our study the failure to detect differential gender-based response may have been caused by the differences in number of men and women recruited or by the young age and childlessness of the participants. Further research is needed to confirm if differences exist and for which groups. 

As in the preferential looking experiment, the comparison with adults in the cuteness task confirms the notion that a high sensitivity to subtle modifications of infantile facial traits may be a process emerging gradually during development: while children judged faces of young individuals and those of adult subjects as similarly cute, adult participants gave higher scores to faces of young subjects. 

### BABY SCHEMA AND FACE PROCESSING

The concept of cuteness not only encompasses the evaluation of specific morphological traits (i.e., cuteness ratings, preference, attractiveness), but also involves a positive/affectionate behavioral response (cute response), which appears to be anticipated by a visual prioritization of – and an attentional bias to – infantile stimuli. The pattern of eye movements is a susceptible index of our attention, motivation and preference and can be modulated by cognitive demands and characteristics of the observed scenes (Henderson, 2003; Isaacowitz, 2006). No studies to date have analyzed whether cuteness perception of different faces involves a different gaze strategy (gaze distribution across key internal facial features, i.e., eyes, nose, and mouth). We predicted that while judging cuteness, gaze patterns would be sensitive to cues specifically related to infant-like characteristic (i.e., big eyes in baby schema) and for the first time we tested such an assumption in children by means of eye tracking. Our results show that, independently of the face viewed, children allocated the majority of fixations and longer viewing time to the eyes, followed by the nose and the mouth. This result is consistent with the evidence of a general oculomotor strategy employed by humans while exploring faces (both human and animals), at least for those sharing similar facial configurations (same components – eyes, mouth, and nose – within a similar spatial arrangement – the nose at the center, the eyes above, and the mouth below; Guo et al., 2010). Nonetheless, our results show that viewing time allocation to the same facial feature is species-dependent and is also sensitive to the degree of baby schema. In particular, after adjusting for the variance in size across different stimuli, we observed that the region of the eyes in high infantile faces attracted longer viewing times than in low infantile faces. The eyes contain critical information about face identity and emotional state (Emery, 2000), attention to the eyes may predict later social development (Jones and Klin, 2013; Wagner et al., 2013), and eye size may affect both aesthetic ratings of and visual preference for human faces (Geldart et al., 1999). Here we suggest that, more than other facial features, they may also be crucial for cuteness perception and associated attentional response. 

Presentation of pictures of different species resulted in a differential distribution of fixations directed to specific face regions: the mouth region in human faces attracted significantly more fixations and longer viewing times than in dog and cat faces, similarly to what was observed in a sample of adults by Guo et al. (2010). As in adults, children’s differential gaze allocation to the mouth could indicate the precocious ability to extract relevant facial information from different species, in particular the importance of the mouth for human visuo-social communication (for fast detection and recognition of subtle facial expressions, Guo et al., 2010) and for human language comprehension (Lewkowicz and Hansen-Tift, 2012). Guo et al. (2010) hypothesized that the failure to detect a differential gaze distribution in viewing dog and cat faces may depend upon a lack of interest and/or perceptual experience in processing subtle emotional cues from dog and cat mouths in their sample of non pet owners. They pointed out that this issue should be addressed by comparing gaze patterns in the viewing of dog/cat faces between pet owners and non-owners. We showed that, at least in children, experience gained by owing a pet, does not influence the distribution of fixations directed at local facial features across species. It cannot be excluded that this effect may be detectable only in adults or in dog/cat experts (e.g., subjects extensively involved in dog training and/or activities, but see Kujala et al., 2012) and further research is warranted. 

### CONCLUSIONS AND FUTURE PERSPECTIVES

Overall our results show that the response to an infantile facial configuration emerges early during development. In children, the baby schema affects both cuteness perception and gaze allocation to infantile stimuli and to specific facial features. Our findings confirm the generalization of the baby schema response to animals, specifically to the most common pet species (dogs and cats). In line with previous research, results from the current study confirm human positive appraisal toward animals that appear only partially dependent on the presence of infantile features and not directly linked with familiarity with them (e.g., pet ownership).

The effect of facial appearance on cuteness and attractiveness was shown to be tied to human interest in infants and motivation to care (Light and Isaacowitz, 2006; Cárdenas et al., 2013). The influence of individual factors in modulating responses to animals should thus be emphasized in future research. Pet ownership may be a measure not highly representative of interest in – and involvement with – animals. In fact, even if they have animals at home, children may not have a great commitment to their daily care. Future studies could thus employ measures more representative of their involvement with household pets, such as frequency of play with and care of pets, attachment to them and frequency of expressed interest (Melson and Fogel, 1996; Archer and Monton, 2011) in a larger sample of children. 

Most importantly, future research is needed to determine the link between overt attention and measures of interest and how both these measures reflect on care-giving behavior. Cuteness judgment may enhance nurturing behavior (Glocker et al., 2009a; Sherman et al., 2009) and has been shown to modulate mother-infant interaction (Langlois et al., 1995) and women’s willingness to adopt a baby (Volk and Quinsey, 2002). This field of analysis has the potential to be successfully translated into the human-animal interaction research, as no studies have explored association between cuteness and adoptability in kennel dogs or cats or to what extent animal appearance influences owner-pet interaction style and care behavior toward pets. 

A more in-depth analysis of human proneness toward animals and its change during development appears of particular importance, especially in the light of the recent advancements in Human-Animal Interaction studies in child psychology research. Animals, especially dogs, are increasingly employed both in educational and therapeutic interventions based on the growing evidence of their positive effects on children’s emotional development (Cirulli et al., 2011; Endenburg and van Lith, 2011). Since attention is one of the key aspects of the learning process (Rochais et al., 2014), interacting with animals may represent a mean for promoting cognitive development (e.g., by enhancing motor skills and ability to follow instructions and by improving memory, Gee et al., 2007, 2009, 2010). Thus future research on the attentional aspect of children’s relationships with pet animals should be encouraged. In addition, the analysis of specific animal characteristics able to elicit emotional/affiliative responses in children could ultimately help develop interventions for children with deficit in the social domain (Berry et al., 2013; O’Haire, 2013) by providing salient and emotionally relevant stimuli (e.g., helping in developing socially interactive robots, see Miklósi and Gácsi, 2012 for the utilization of animals as a model for social robotics).

More detailed knowledge of the factors underlying children’s attraction to animals will also facilitate educational programs aimed at minimizing risk factors inherent in children-animal encounters, especially in consideration of dog bite incidents. As young children under the age of 7 are most at risk of a serious dog bite injury, often after an interaction they initiated themselves, we need to investigate the causes further. Attractiveness to animals may be one of the causal factors behind the frequent and sometimes tragic involvement of young children in dog bite incidents. Interestingly, our results show children paying less attention to the mouth region in dog stimuli and this information is crucial insofar as it can help to direct educational efforts to teach children about safe behavior with dogs (since more severe aggression signals in dogs like showing teeth, snarling and growling are displayed in the mouth region, Shepherd, 2002; Meints and de Keuster, 2009). 

This research is a first and significant step toward characterizing both cognitive (attention) and psychological (overt preference) mechanisms underlying human attraction to infants (and infant-like stimuli including animals). Procedures and stimuli as used in this study can easily be further applied in psychological studies, as well as in fMRI and eye-tracking research and provide a wide-ranging platform to deepen our knowledge of the mechanisms and factors that promote human caregiving behavior.

## Conflict of Interest Statement

The authors declare that the research was conducted in the absence of any commercial or financial relationships that could be construed as a potential conflict of interest.

## Footnotes

1 http://www.thinkstockphotos.it

2 http://www.qualtrics.com
